# Supplementary material for: Photodynamic inactivation of multidrug-resistant strains of Klebsiella pneumoniae and Pseudomonas aeruginosa in municipal wastewater by tetracationic porphyrin and violet-blue light: The impact of wastewater constituents
Source: PLoS One. 2023 Aug 15;18(8):e0290080. doi: 10.1371/journal.pone.0290080 (PMC10427015; doi:10.1371/journal.pone.0290080)
Supplement: S1 Table — (PDF) [file pone.0290080.s001.pdf]

Parameter tested	Obtained results		Parameter tested	Obtained results	
	raw municipal wastewater	autoclaved municipal wastewater		raw municipal wastewater	autoclaved municipal wastewater
Conductivity (µS/cm)	18242	18920	Chloride (mg/L)	6184	6073
pH	7.43	7.68	Ammonia (mg/L as N)	17.6	18.2
Visible waste matter	small floating particles	small floating particles	Nitrites (mg/L as N)	0.086	0.004
Odor	by fecal matter	by fecal matter	Nitrates (mg/L as N)	0.3	0.22
Dissolved oxygen (mg/L as O_2)	5.6	5	Kjedahl nitrogen (mg/L as N)	25.2	26.7
Total solid waste (mg/L)	12534	6539	Total nitrogen (mg/L as N)	25.6	26.9
Suspended matter (mg/L)	98	22.5	Orthophosphates (mg/L as P)	1.28	0.947
Total fats and oils (mg/L)	14.6	< 5.0	Total phosphorus (mg/L as P)	2.92	1.72
Nonionic detergents (mg/L)	< 0.20	< 0.20	Phenols (mg/L)	0.017	0.013
Anionic detergents (mg/L)	1.58	1.06	Zn (mg/L)	0.22	0.253
Cationic detergents (mg/L)	0.49	0.307	Cd (mg/L)	0.0006	0.0002
COD* (mg/L per O_2)	280	189	Cr (mg/L)	0.038	0.009
BOD5** (mg/L per O_2)	130	50	Pb (mg/L)	0.009	0.002
AOX***	0.082	0.06	Hg (mg/L)	0.0003	< 0.00025
Color (mg/L Pt)	62	58			

*COD-Chemical Oxygen Demand; **BOD5-Biochemical Oxygen Demand; ***AOX-Adsorbable Organic Halides
