# Supplementary material for: Photodynamic inactivation of multidrug-resistant strains of Klebsiella pneumoniae and Pseudomonas aeruginosa in municipal wastewater by tetracationic porphyrin and violet-blue light: The impact of wastewater constituents
Source: PLoS One. 2023 Aug 15;18(8):e0290080. doi: 10.1371/journal.pone.0290080 (PMC10427015; doi:10.1371/journal.pone.0290080)
Supplement: S2 Table — (PDF) [file pone.0290080.s002.pdf]

**Bacterial strains**	*Pseudomonas aeruginosa* ATCC 27853	*Klebsiella pneumoniae* ATCC 700603	*K. pneumoniae* NCTC 13442
**Antibiotics**	**Susceptibility to antibiotics***		
Gentamicin	R	S	R
Amikacin	S	R	S
Meropenem	R	R	R
Amoxicillin/Clavulanic acid	R	R	R
Ceftazidime	I	R	I
Ceftriaxone	ND	R	S
Cefuroxime	ND	R	S
Cefepime	I	R	R
Imipenem	I	R	S
Cefoxitin	ND	R	S
Ciprofloxacin	I	S	R
Ertapenem	R	R	R
Piperacillin/tazobactam	I	R	R

* R - resistant; I - indifferent; S - Sensitive; ND - not done
