# Supplementary material for: Photodynamic inactivation of multidrug-resistant strains of Klebsiella pneumoniae and Pseudomonas aeruginosa in municipal wastewater by tetracationic porphyrin and violet-blue light: The impact of wastewater constituents
Source: PLoS One. 2023 Aug 15;18(8):e0290080. doi: 10.1371/journal.pone.0290080 (PMC10427015; doi:10.1371/journal.pone.0290080)
Supplement: S3 Table — (PDF) [file pone.0290080.s003.pdf]

**TMPyP4**	**DARK**	**irr. λ = 394 nm; 20 mW/cm^2; 12 J/cm^2**
P. aeruginosa	>50 µM	25 µM
K. pneumoniae	>50 µM	3.125 µM
K. pneumoniae OXA-48	50 µM	6.25 µM
